# 3D-Printed Load Cell Using Nanocarbon Composite Strain Sensor

**DOI:** 10.3390/s21113675

**Published:** 2021-05-25

**Authors:** Kwan-Young Joung, Sung-Yong Kim, Inpil Kang, Sung-Ho Cho

**Affiliations:** 1 Department of Electronic Engineering, Hanyang University, Seoul 04763, Korea; j6044@kitech.re.kr; 2 Department of Innovative Smart Manufacturing R&D, Korea Institute of Industrial Technology, Cheonan 31056, Korea; 3 Department of Mechanical and Design Engineering, Pukyong National University, Busan 48513, Korea; ksy1357@pukyong.ac.kr (S.-Y.K.); ipkang@pknu.ac.kr (I.K.)

**Keywords:** load cell, carbon nanotube, strain sensor, 3D printing, piezoresistivity

## Abstract

The development of a 3D-Printed Load Cell (PLC) was studied using a nanocarbon composite strain sensor (NCSS) and a 3D printing process. The miniature load cell was fabricated using a low-cost LCD-based 3D printer with UV resin. The NCSS composed of 0.5 wt% MWCNT/epoxy was used to create the flexure of PLC. PLC performance was evaluated under a rated load range; its output was equal to the common value of 2 mV/V. The performance was also evaluated after a calibration in terms of non-linearity, repeatability, and hysteresis, with final results of 2.12%, 1.60%, and 4.42%, respectively. Creep and creep recovery were found to be 1.68 (%FS) and 4.16 (%FS). The relative inferiorities of PLC seem to originate from the inherent hyper-elastic characteristics of polymer sensors. The 3D PLC developed may be a promising solution for the OEM/design-in load cell market and may also result in the development of a novel 3D-printed sensor.

## 1. Introduction

Load cells convert forces such as pressure or load into electrical signals. They are some of the most widely used sensors in our daily lives, with industrial, automotive, and measurement applications. Load cell types include hydraulic, pneumatic, piezoelectric, capacitance, and strain gauge, with strain gauge being the most common [1]. Within the internal structure of a strain gauge load cell (called a flexure), a structure such as a beam or a membrane is deformed under the loading conditions. These load cells commonly measure the deformation of the flexure with conventional foil or semiconductor strain gauges. A typical foil strain gauge uses a piezoresistive mechanism based on electrical resistance fluctuations due to changes in its length and cross-section under the load. The load cell is generally designed to meet the desired specifications of a specific structure, so that its performance is closely related to the characteristics of the strain gauge. A foil strain gauge is manufactured by processing the alloy material into a fine pattern using an etching method; its sensitivity, which is represented by the gauge factor, determines the alloy properties [2]. A foil strain gauge has low sensitivity and weak durability. A semiconductor strain gauge has high sensitivity with a larger output, which can be used to develop high-performance load cells [3]. A semiconductor strain gauge is manufactured using a silicon crystal cutting process; however, its brittleness makes it less producible and less durable, which limits its suitability for many industrial applications [4].

Newly emerging materials may be used to develop sensors that overcome some of these problems [5,6]. Nanocarbon allotrope, such as carbon nanotubes (CNT) and graphene, may enable the development of new load cells with higher sensitivity, better mechanical strength, and simpler machining [7,8,9]. Nanocarbon allotrope consisting of continuous carbon atoms have high mechanical strength and excellent electrical conductivity, making them suitable for use in new smart nanocomposite materials with ultra-light, high-strength conductivity [10,11,12,13,14]. A nanocarbon-based composite may be an ideal sensory material because of its electrical and mechanical properties [15,16]. When used as fillers in the composite, nanocarbon allotropes improve the mechanical strength and create conductive paths in the composite [17].

When a nanocarbon-based composite is deformed, the composite’s electrical properties change due to internal conductive conditions in the process of piezoresistivity. Therefore, we developed nanocarbon composite strain sensors (NCSS) using a nanocarbon piezoresistive composite (NCPC), which showed similar performance to commercial foil strain gauges [18,19]. Under compression loading, NCPC internal nanofillers move closer to each other and thus increase their electrical conductivity, thereby reducing the electrical resistance of the NCPC. Conversely, NCPC electrical resistance is increased under tensile load due to its broken internal conductive networks. NCPC can be used to develop new types of strain sensors and load cells because of its promising material characteristics, which are different from conventional metal or ceramic materials. Specifically, we focus on the beneficial machining properties of NCPC, which allow various load cell structures to be easily manufactured with a three-dimensional (3D) printing process. A 3D printing process using NCPC paint or filaments may be used to fabricate novel load cells.

With 3D printing technology, a device can be easily designed and manufactured using polymeric materials [20,21,22,23]. 3D printing combined with NCPC has been used in the development of sensors. Kim et al. fabricated 3D-printed multiaxial force sensors using CNT/TPU filaments. In this study, the electrical characteristics of the 3D-printed sensor were demonstrated through various experiments, such as the three-point bending and a repeated bending cycle test [24]. Christ et al. fabricated TPU/MWCNT strain sensors using 3D printing and evaluated their electrical properties [25]. Al-Rubaiai et al. fabricated stretchable strain sensors using 3D printing for wind sensing [26]. Stano et al. fabricated load cell structures and sensor parts with an FFF method using TPU and CNT filaments [27]. 3D-printed strain-gauge microforce sensors were recently developed. Qu et al. demonstrated the effectiveness of 3D-printed force sensors in a proof-of-concept demonstration. In their study, 3D-printed microforce sensors with micronewton sensing resolutions were fabricated by a fused deposition method (FDM) or stereo lithography (SLB) method. Only the flexure was fabricated with a 3D printing process; commercial strain gauges were used to develop the force sensors [28].

A load cell using the NCSS for its sensory part may be a promising solution in the development of a novel 3D-printed sensor. Despite the fact that many studies have investigated 3D printing technology for sensors, problems arising from the use of polymer materials still exist. Polymer-based 3D printing sensors have some unstable electrical characteristics such as hysteresis, recovery, and response time due to the viscoelastic properties of polymer. To overcome these problems, it is necessary to study the characteristics of 3D printing polymer sensor systematically. Since the performance of the sensor directly relates to its material property and structural forming, the aim of our contribution is to study the piezoresistive behavior of NCPC when it forms a sensing structure such as a flexure of load cell fabricated by a polymer-based 3D printing process. A miniaturized load cell with a simple beam flexure was developed to be easily fabricated using a 3D printing process. A finite element method (FEM) was conducted to design the load cell flexure, and piezoresistive paint made of MWCNT/epoxy composite was used to fabricate the sensor part of the flexure. The load cell was fabricated using a low-cost LCD-type 3D printer. The sensing performance of the printed load cell (PLC) was studied in terms of loading voltage output characteristics.

## 2. 3D Printed Load Cell Design and Fabrication

### 2.1. 3D Printed Load Cell Design

A load cell generally requires various flexure structure types to optimize or maximize its sensor performance. Although the fabrication of some flexures was traditionally limited to conventional MEMS processes if they had complicated structures, 3D printing processes using NCPC are free from such problems. If a 3D printer filament is developed with NCPC, the complex flexure can be manufactured by 3D-printing additive processes. Furthermore, it may be able to fabricate an integral load cell with a flexure that includes the sensing parts. In a previous study, we reported on a miniaturized pressure sensor built by a 3D FDM printer with an ABS filament, and its sensing part was made of NCPC paint [29]. The cantilever sensing part of the pressure sensor showed a performance improvement of approximately 200% with linear output voltage compared to a bulk-sensing part without specific structural features. Therefore, we planned to develop a 3D-printed load cell based on the previous works and to hire epoxy resin to build a more tough body than the previous one. We benchmarked the specifications of a commercial load cell to develop a miniaturized 3D-printed load cell. The scheme of the miniaturized load cell involved a flexure that had a beam with two fixed ends, which could be manufactured using a 3D printer to allow for simple fabrication.

The flexure dimensions were based on general resin strength (E = 3.8GPa), maximum load, and PLC size. The PLC diameter was 30 mm. A maximum load (P) of 2 kg was tentatively selected based on the fragility of the resin. Considering the interior space of the PLC, the final beam dimensions were 17.3 mm × 2.6 mm × 1 mm (length × width × thickness).

**Figure 1:**
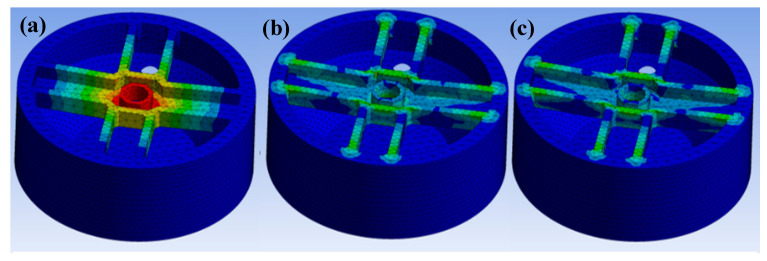
Finite element method (FEM) analysis using ANSYS: (**a**) deformation analysis; (**b**) strain analysis; and (**c**) stress analysis.

The flexure structural behavior was simulated by an FEM analysis using ANSYS to determine the maximum beam deflection and load, with the results depicted in Figure 1 and Table 1. After applying 2 kg to the flexure, ANSYS showed a maximum stress of 55 MPa around the spoke, which just exceeded the resin epoxy tensile yield strength (54.6 MPa). 3D printed parts are not 100% solid and their internal structure significantly affects their mechanical properties. Therefore, to design the flexure, we hired a safety factor (1.5) considering the structural uncertainties. The rated capacity of the PLC was less than 1.3 kg considering the safety factor and the experimental load criterion was determined to be less than 1 kg by a sensor characterization test. We concluded that the beam design was acceptable based on the strength of the 3D-printed resin and the maximum load. We plan to conduct a future study on the optimized flexure shape using computational structural analysis to improve the performance of the load cell. 

### 2.2. Load Cell Fabrication Using 3D Printing Process

**Figure 2:**
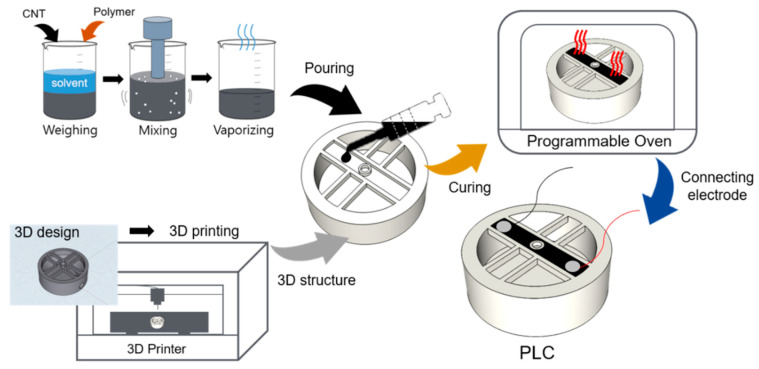
Process to fabricate the printed load cell (PLC) structure and the nanocarbon piezoresistive composite flexure.

**Figure 3:**
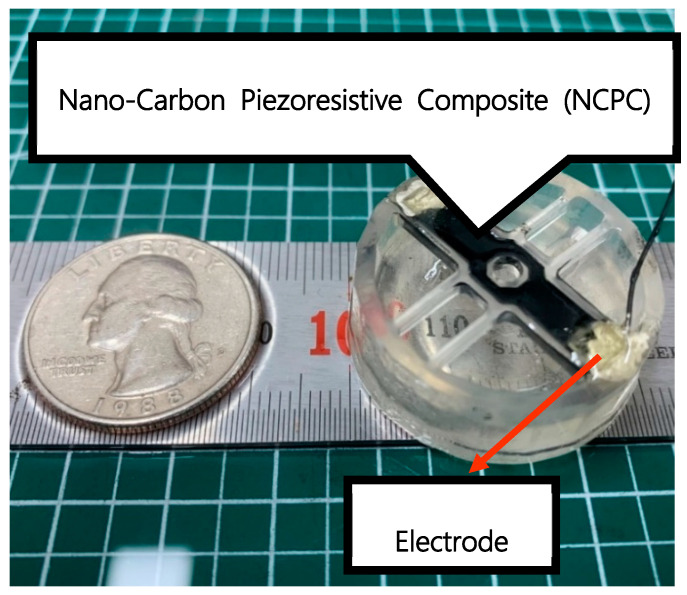
The 3D PLC using nanocarbon composite strain (diameter: 30 mm, height: 10 mm, resistance: 920 kΩ).

To fabricate the sensing part on the flexure of the load cell, we created a piezoresistive paint using the NCPC process depicted in Figure 2. The load cell body was built using a popular and low-cost 3D printer (Creality 3D LD-002R) with 4thWave Resin, as shown in Figure 3; 4th Wave Resin is a UV tough resin and its mechanical properties are better than ABS; thus, it was mainly used in this experiment. It has mechanical properties of 85 MPa, tensile strength 41.3 MPa, flexural strength 47 MPa, elastic modulus 570.8, elongation 9.2, and shrinkage 6.9% [30]. The 3D printer used an LCD technique with a 0.4 mm nozzle for UV resin, which provided an in-plane printing resolution of 0.075 mm and a vertical printing resolution of 0.05 mm. The sensor structure was built by 100% infill by the 3D printer and the flexure was built by slender and thin four-way support beams to withstand the target load and to obtain a maximum bending effect.

The sensory part of the load cell was fabricated by NCSS made of NCPC. We blended 0.5 wt% MWCNT (Hanhwa Chemical Co., Seoul, Korea, CM-280) and 99.5 wt% epoxy (Kukdo Chemical Co., Seoul, Korea, YD-128) to make the NCPC paint. The NCPC paint was prepared using a similar process to the one described in our previous study. The ratio of the MWCNT and polymer was selected according to the percolation threshold experiment from our previous works, which had a gauge factor of approximately 2 [18].

The NCPC paint was injected to groove on the flexure and cured to form the sensing part of the PLC. After the curing process for the sensing part, two electrical wires were connected at the end point of the NCPC layer using silver conducting epoxy (ElcoatCleantech, Ansan, Korea, Elcoat P-100) to pick up a piezoresistive signal.

## 3. Experimental 3D-Printed Load Cell Sensing Characteristics

### 3.1. Experimental Setup

**Figure 4:**
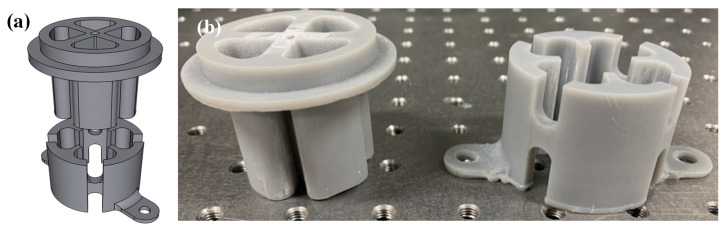
The 3D printed jig for loading test: (**a**) CAD draft; and (**b**) the fabricated top and bottom part of the Jig.

**Figure 5:**
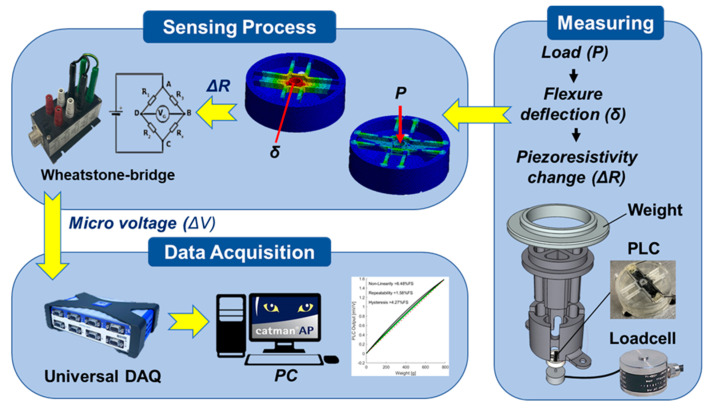
Schematic illustration of the 3D printing load cell sensing characteristics measurement setup.

To evaluate the PLC output characteristics for load changes, its voltage outputs were compared to a commercial industrial miniature load cell (SETech, Seoul, Korea, YC33-50K). A test jig was fabricated with the 3D printer to add weight on the two overlapped load cells at the same time, as illustrated in Figure 4. The 3D PLC voltage responses were compared to the reference load cell; the responses were measured by general signal processing, as depicted in Figure 5.

**Figure 6:**
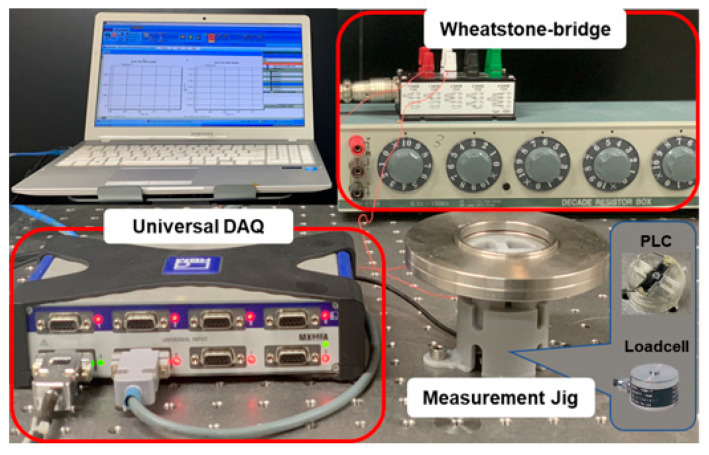
Experimental apparatus with a 3D printed jig and a data acquisition system.

As the flexure was bent due to an external load, the deflection (δ) induced a resistance change (ΔR) in the NCPC sensing part on the PLC, which was converted to micro voltage output through a Wheatstone quarter-bridge. The Wheatstone bridge can compensate for temperature effects; a full bridge is commonly used to cancel the signals caused by extraneous forces. In this study, we used a quarter-bridge to monitor the basic responses of the PLC without any compensation. The voltage output from the Wheatstone bridge was amplified and filtered via a data acquisition system (QuantumX-MX-840, HBM Co., Marlborough, MA, USA) with a 50 Hz sampling frequency and 5 Hz low-pass filter. Figure 6 shows the experimental apparatus with the jig and data acquisition system.

### 3.2. 3D PLC Sensing Characteristics

The PLC sensing properties were characterized for indoor conditions based on the standard calibration process of load cells while considering experimental constraints in an early laboratory-level development stage. High-precision standard weights were applied to the load cells by the 3D-printed jig and voltage outputs were measured. However, due to the limitations of the 3D-printed jig precision, a reference load cell was also used to evaluate the loading conditions. The PLC outputs were measured five times after reaching steady state and were averaged after eliminating the maximum and minimum data points.

**Figure 7:**
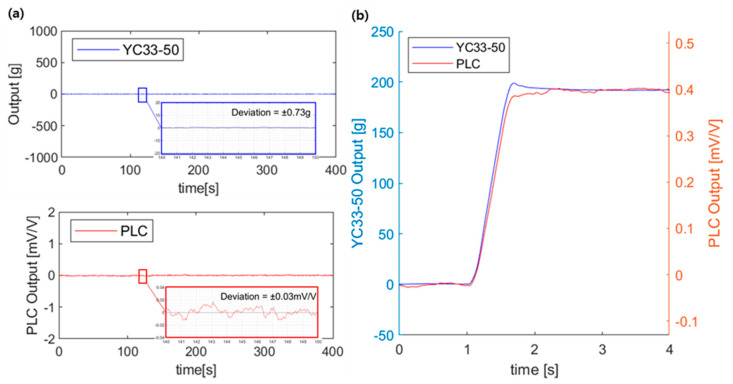
Response profiles of the 3D PLC and the commercial load cell: (**a**) voltage outputs under no loading; and (**b**) voltage outputs under a static load 0.2 kg (step response).

For the sensor characterization, the PLC response stability was first evaluated by measuring the voltage outputs under no loading and under a static load (0.2 kg), as shown in Figure 7. The PLC voltage output showed a stable response with some deviation (±0.03 mV/V, ±20 g). A noisy response has been reported for NCPC due to a resistance drift of the composite resistance originating from the inherent electrical changing properties of CNTs based on the surrounding environment [31]. However, the electrical signal stability of PLC with NCSS is sufficient for engineering applications, as we reported previously [18,19]. The response times of the two load cells, presented in Figure 7b, were nearly identical.

**Figure 8:**
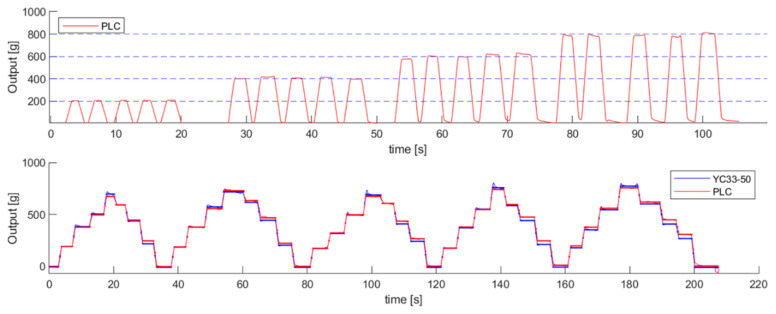
A successive loading test of the PLC compared with YC-33.

A series of weights was successively added to the PLC to qualitatively determine its characteristics; the responses are shown in Figure 8. Reproducible responses were exhibited by the PLC, which showed a drift problem similar to other polymer-based strain sensors. The PLC response was initially well matched with the reference load cell; however, after some time, gaps were found due to the drift. The drift seemed to be caused by hysteresis, because it mostly happened during unloading and later. Other studies have reported that the drift response is affected by the inherent characteristics of polymers [32,33].

**Figure 9:**
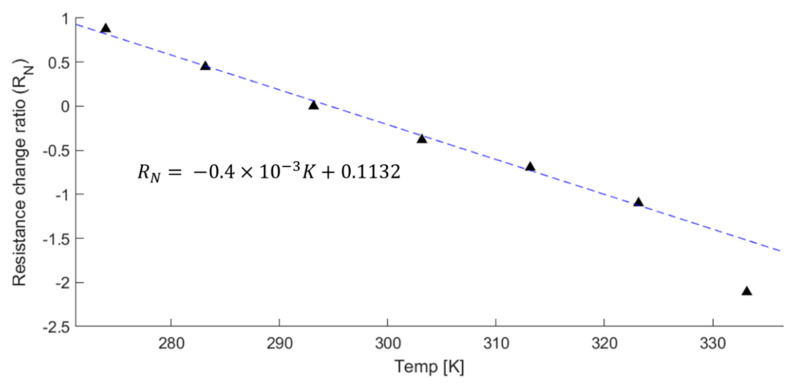
Temperature dependence of the PLC.

The resistance of NCPC changes with temperature fluctuations [34]. These changes in resistance may affect the output voltage from the temperature of the PLC. Figure 9 shows the change ratio of the resistance in relation to the temperature.

The effect of temperature on the output characteristics of a traditional load cell is the biggest factor impairing accuracy. Thus, the PLC output voltage also needs to be adjusted relative to temperature changes. The temperature dependency of PLC resistance tends to change linearly to 50 °C, after which it remarkably changes. The temperature sensitivity was found to be −0.4 × 10^−3^ K^−1^, which was comparable to other 0.5 wt% MWCNT/epoxy cases that had temperature sensitivities of −0.6 × 10^−3^ K^−1^ [35]. Considering the polymeric characteristics of PLC, its operating temperature can be restricted beyond the allowable temperature range (~70 °C) of industrial load cells.

**Figure 10:**
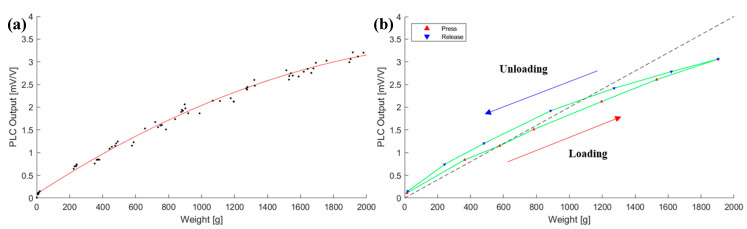
Loading/unloading test of the PLC to the maximum analytical load, 2 kg: (**a**) hysteresis characteristic; and (**b**) 5 time measurements.

The output voltages were measured to evaluate the linearity and sensitivity of PLC. To begin the characterization test of the PLC, we applied loads up to the theoretical maximum (2 kg) to investigate its behavior, as depicted in Figure 10.

**Figure 11:**
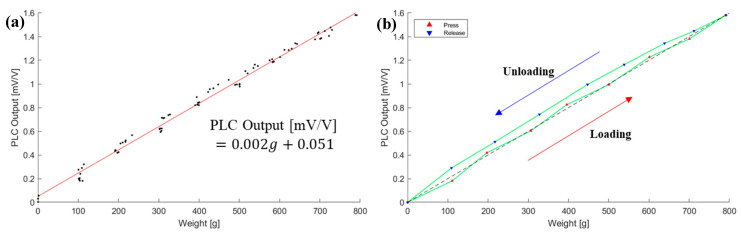
Loading/unloading test of the PLC to the rated load, 0.8 kg: (**a**) 5 time measurements; and (**b**) hysteresis characteristic.

The PLC held up to the analytical maximum load of 2 kg. However, it showed nonlinearly for a loaded response above the 1 kg range and also exhibited hysteresis. From this test, the rated load span was conservatively determined to be 0.8 kg to protect the flexure and to obtain a linear response. The PLC characteristics were tested again within the rated load range, as shown in Figure 11.

**Figure 12:**
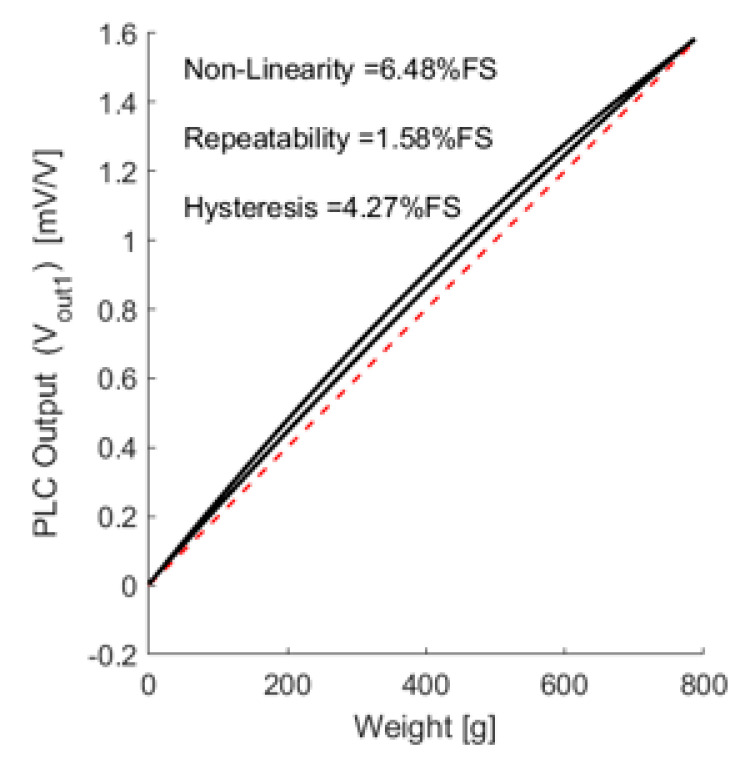
PLC performance non-compensated characterization.

At the rated load range, the PLC showed a linear response, including hysteresis. The PLC output was evaluated to the most common value of 2 mV/V. The PLC performance parameters were derived based on this test, as depicted in Figure 12.

**Figure 13:**
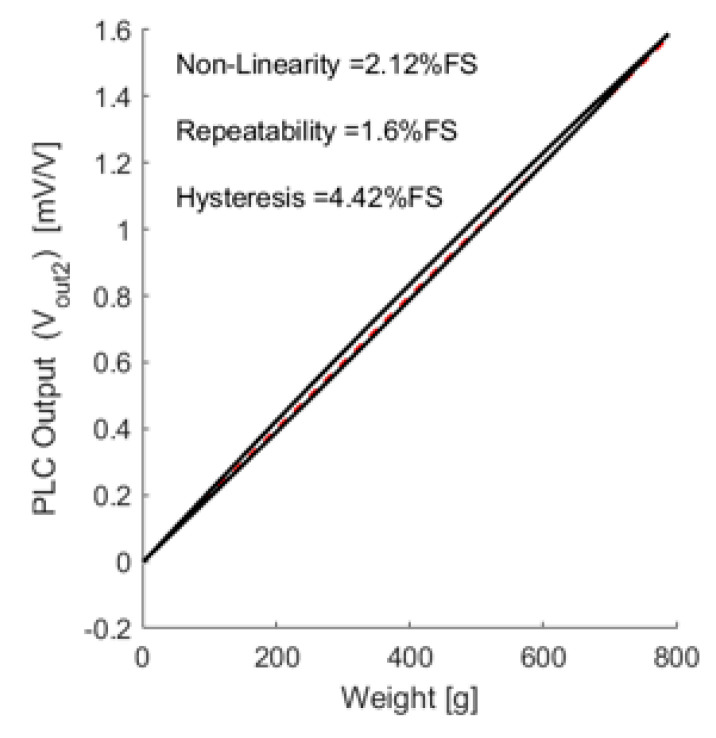
The calibrated PLC performance characterization.

The first preliminary evaluation (*V*_*out*_1__) is presented in Figure 11. It was fitted to a second-order polynomial, shown in Equation (1), in order to compensate for the PLC output. The compensated PLC output is presented in Figure 13.

(1) $$V_{out_{2}}=0.09{V^{2}}_{out_{1}}+0.87V_{out_{1}}-0.02$$

After the compensation, the linearity improved. The end point linearity was 2.12% for full-scale non-linearity, which was less than that of a conventional load cell.

**Figure 14:**
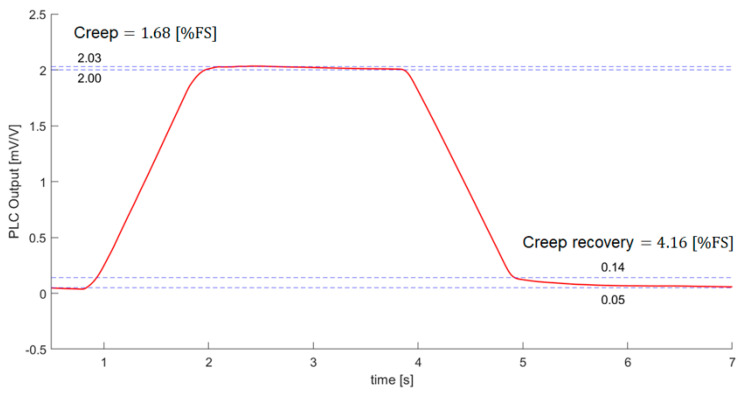
PLC creep test under maximum rated load 0.8 kg.

To test the creep, we only considered time and tested the load cell output voltage under the maximum rated load of 0.8 kg at room temperature without considering other environmental conditions. Figure 14 shows the creep test. Because the flexure was made of epoxy resin, creep was expected to occur when a load was applied for a period of time. Creep may be more considerable for PLC than for a commercial a load cell made of metal.

An international standard, OIML R 60, recommends that creep should be evaluated using data measured for more than 30 min [36]. We also currently suffer difficulties to obtain long term signal stability due to the viscoelastic properties of polymer. Therefore, only limited steady-state data were available to test the creep of PLC. We did a simple creep test to remove the load applied to the PLC after applying a load of 0.8 kg to the PLC in the no-load state. The creep and creep recovery values can be obtained as “(peak value-stable value)/rated output”. Creep and creep recovery were found to be 1.68 (%FS) and 4.16 (%FS). The PLC creep behavior was not comparable to that of a commercial load cell, but it can be improved by compensation.

Based on the parameters conceptually evaluated in this preliminary laboratory investigation (Table 2), the performance of the PLC does not yet meet industrial market criteria, which require less than 1% [1]. The inferior results found for the PLC may be due to the inherent hyper elastic characteristics of polymer sensors. A future study is planned to investigate approaches to improve PLC performance, including compensation for drift, hysteresis, and temperature based on characteristic models.

## 4. Conclusions

The development of a 3D PLC was studied using a nanocarbon composite strain sensor (NCSS) and a 3D printing process. A miniaturized load cell with a simple beam flexure could be easily fabricated using a 3D printing process. To design the sensory part of the load cell, the structural behaviors of the flexure were analyzed using a finite element method. Its loading capacity was conservatively determined to be less than 1 kg based on the strength of the resin epoxy.

The miniature load cell was fabricated using a low-cost LCD-based 3D printer with UV resin. An NCSS composed of NCPC with 0.5 wt% MWCNT/epoxy was used to create the flexure. The sensing performance of the PLC was studied in terms of its voltage output characteristics under a 0.8 kg load, which was the linear loading response range. A zero-balance voltage output was stable with some deviation (±0.01 mV/V, ±20 g) due to the inherent noisy response of NCPC. The response time was nearly identical to that of a commercial load cell. The PLC showed drift problems in a successive loading/unloading test, as well as temperature dependence (−0.4 × 10^−3^ K^−1^).

PLC performance was evaluated under a rated load range; its output was equal to the common value of 2 mV/V. The performance was also evaluated after calibration in terms of non-linearity, repeatability, and hysteresis, with final results of 2.12%, 1.60%, and 4.42%, respectively; these results do not yet meet industrial market criteria. Creep and creep recovery were found to be 1.68 (%FS) and 4.16 (%FS). PLC creep behavior was not comparable to that of a commercial load cell, but it can be improved by compensation. The relative inferiorities of PLC seem to originate from the inherent hyper elastic characteristics of polymer sensors. Thus, a future study is planned to investigate compensation for drift, hysteresis, and temperature based on characteristic models.

Because of its excellent manufacturing and piezoresistive properties, an NCSS made of NCPC was investigated for use in the sensory part of a 3D PLC’s flexure. Our results indicate that NCPC and the 3D printing process may be promising tools in the development of novel load cells. A customized circuit and compensation model may be required to achieve an accurate performance. To develop a completely 3D-printed load cell, we are investigating a 3D-printed filament made of NCPC that allows both the flexure and sensory parts to be fabricated simultaneously through the 3D printing process. Further study of the flexure design is planned to determine a customized load capacity. We are also planning a sensing compensation study to obtain reliable signals based on the characteristic model, which will improve the NCPC-based 3D PLC for practical applications. The 3D PLC developed in this study may be a promising solution for the OEM/design-in load cell market and may also result in the development of a novel 3D-printed sensor.

## Figures and Tables

**Table 1:** Finite element method (FEM) analysis result.

Max. Load (kg)	Max. Deflection (mm)	Max. Strain (%)	Max. Stress (MPa)
2	0.29	2	55

**Table 2:** PLC performance.

Parameter	Non-Linearity [%]	Repeatability [%]	Hysteresis [%]
Evaluation (1st)	6.48	1.58	4.27
Evaluation (compensated)	2.12	1.60	4.42
Market requirement [1]	0.25~1	0.1~0.5	0.25~1

## Data Availability

Data available in a publicly accessible repository.

## References

[1] PCB Load & Torque Division (2014). Load Cell Handbook: A Technical Overview and Selection Guide.

[2] OMEGA. https://kr.omega.com/prodinfo/loadcells.Html.

[3] Higson G.R. (1964). Recent Advances in Strain Gauges. J. Sci. Instrum..

[4] Yang S., Lu N. (2013). Gauge Factor and Stretchability of Silicon-on-Polymer Strain Gauges. Sensors.

[5] Mangayil R., Rajala S., Pammo A., Sarlin E., Luo J., Santala V., Karp M., Tuukkanen S. (2017). Engineering and Characterization of Bacterial Nanocellulose Films as Low Cost and Flexible Sensor Material. ACS Appl. Mater. Interfaces.

[6] Lim H.-R., Kim H.-S., Qazi R., Kwon Y.-T., Jeong J.W., Yeo W.-H. (2019). Advanced Soft Materials, Sensor Integrations, and Applications of Wearable Flexible Hybrid Electronics in Healthcare, Energy, and Environment. Adv. Mater..

[7] Pan S., Pei Z., Jing Z., Song J., Zhang W., Zhang Q., Sang S. (2020). A Highly Stretchable Strain Sensor Based on CNT/graphene/Fullerene-SEBS. RSC Adv..

[8] Ali A., Ali F., Irfan M., Muhammad F., Glowacz A., Antonino-Daviu J.A., Caesarendra W., Qamar S. (2020). Mechanical Pressure Characterization of CNT-Graphene Composite Material. Micromachines.

[9] Sheng N., Ji P., Zhang M., Wu Z., Liang Q., Chen S., Wang H. (2021). High Sensitivity Polyurethane-Based Fiber Strain Sensor with Porous Structure via Incorporation of Bacterial Cellulose Nanofibers. Adv. Electron. Mater..

[10] Kang I., Schulz M.J., Kim J.H., Shanov V., Shi D. (2006). A Carbon Nanotube Strain Sensor for Structural Health Monitoring. Smart Mater. Struct..

[11] Salvado R., Lopes C., Szojda L., Araujo P., Gorski M., Velez F.J., Castro-Gomes J., Krzywon R. (2015). Carbon Fiber Epoxy Composites for Both Strengthening and Health Monitoring of Structures. Sensors.

[12] Hu N., Itoi T., Akagi T., Kojima T., Xue J., Yan C., Atobe S., Fukunaga H., Yuan W., Ning H. (2013). Alamusi Ultrasensitive Strain Sensors Made from Metal-Coated Carbon nanofiller/Epoxy Composites. Carbon.

[13] Ferreira A., Mendez S.L. (2016). Piezoresistive Response of Spray-Printed Carbon nanotube/Poly(vinylidene Fluoride) Composites. Compos. Part B Eng..

[14] Le T.H., Lee D.H., Kim J.H., Park S.J. (2020). Polypyrrole/Graphene Quantum Dot Composites as a Sensor Media for Epinephrine. J. Nanosci. Nanotechnol..

[15] Park M., Kim H., Youngblood J.P. (2008). Strain-Dependent Electrical Resistance of Multi-Walled Carbon Nanotube/Polymer Composite Films. Nanotechnology.

[16] Li C., Thostenson E.T., Chou T.-W. (2007). Dominant Role of Tunneling Resistance in the Electrical Conductivity of Carbon nanotube–based Composites. Appl. Phys. Lett..

[17] Park S.-H., Hwang J., Park G.-S., Ha J.-H., Zhang M., Kim D., Yun D.-J., Lee S., Lee S.H. (2019). Modeling the Electrical Resistivity of Polymer Composites with Segregated Structures. Nat. Commun..

[18] Choi G., Lee J.W., Cha J.Y., Kim Y.-J., Choi Y.-S., Schulz M.J., Moon C.K., Lim K.T., Kim S.Y., Kang I. (2016). A Spray-On Carbon Nanotube Artificial Neuron Strain Sensor for Composite Structural Health Monitoring. Sensors.

[19] Kim S.Y., Choi B.G., Baek W.K., Park S.H., Park S.W., Shin J.W., Kang I. (2019). Impact Paint Sensor Based on Polymer/Multi-Dimension Carbon Nano Isotopes Composites. Smart Mater. Struct..

[20] Giovanni P., Gabriele N., Gianmarco G., Marinella L., Stefano T. (2015). Conductive 3D Microstructures by Direct 3D Printing of polymer/Carbon Nanotube Nanocomposites via Liquid Deposition Modeling. Compos. Part A Appl. Sci. Manuf..

[21] Zhang D., Chi B., Li B., Gao Z., Du Y., Guo J., Wei J. (2016). Fabrication of Highly Conductive Graphene Flexible Circuits by 3D Printing. Synth. Met..

[22] John M.H., Godfrey S., Kim J.W., Roberto J.C., Russel A.W., Christopher J.S., Brian W.G., Dennis C.W., Emilie J.S. (2016). 3-D Printing of Multifunctional Carbon Nanotube Yarn Reinforced Components. Addit. Manuf..

[23] Rafael M.C., Cristiane K., Raquel G.R., Pãmyla L.S., Diego P.R., Paulo R.O., Bruno C.J., Juliano A.B., Eduardo M.R., Rodrigo A.A.M. (2020). Additive-Manufactured (3D-Printed) Electrochemical Sensors: A Critical Review. Anal. Chim. Acta.

[24] Kim K., Park J., Suh J.-H., Kim M., Jeong Y., Park I. (2017). 3D printing of multiaxial force sensors using carbon nanotube (CNT)/thermoplastic polyurethane (TPU) filaments. Sens. Actuators A Phys..

[25] Christ J.F., Aliheidari N., Pötschke P., Ameli A. (2018). Bidirectional and Stretchable Piezoresistive Sensors Enabled by Multimaterial 3D Printing of Carbon Nanotube/Thermoplastic Polyurethane Nanocomposites. Polymers.

[26] Al-Rubaiai M.K., Tsuruta R., Gandhi U., Wang C., Tan X. (2019). A 3D-Printed Stretchable Strain Sensor for Wind Sensing. Smart Mater. Struct..

[27] Stano G., Nisio A.D., Lanzolla A., Percoco G. (2020). Additive Manufacturing and Characterization of a Load Cell with Embedded Strain Gauges. Precis. Eng..

[28] Qu J., Wu Q., Clancy T., Fan Q., Wang X., Liu X. (2020). 3D-Printed Strain-Gauge Micro Force Sensors. IEEE Sens. J..

[29] Kim S.Y., Kang I. (2017). A Study on the Development of a Novel Pressure Sensor Based on Nano Carbon Piezoresistive Composite by Using 3D Printing. Trans. Korean Soc. Mech. Eng. A.

[30] 4thwave. https://4thwave3d.com/product/Resin/.

[31] Kordas K., Mustonen T., Toth G., Jantunen H., Lajunen M., Soldano C., Talapatra S., Kar S., Vajtai R., Ajayan P.M. (2006). Inkjet Printing of Electrically Conductive Patterns of Carbon Nanotubes. Small.

[32] Christ J.F., Aliheidari N., Ameli A., Pötschke P. (2017). 3D Printed Highly Elastic Strain Sensors of Multiwalled Carbon Nano-tube/Thermoplastic Polyurethane Nanocomposites. Mater. Des..

[33] Oliveri A., Maselli M., Lodi M., Storace M., Cianchetti M. (2018). Model-Based Compensation of Rate-Dependent Hysteresis in a Piezoresistive Strain Sensor. IEEE Trans. Ind. Electron..

[34] Dehghani S., Moravvej-Farshi M.K., Sheikhi M.H. (2012). Temperature Dependence of Electrical Resistance of Individual Carbon Nanotubes and Carbon Nanotubes Network. Mod. Phys. Lett. B.

[35] Neitzert H.C., Vertuccio L., Sorrentino A. (2011). Epoxy/MWCNT Composite as Temperature Sensor and Electrical Heating Element. IEEE Trans. Nanotechnol..

[36] International Organization of Legal Metrology (2000). Metrological Regulation for Load Cells.

